# Epicardial adipose tissue as a prognostic marker in acute pulmonary embolism

**DOI:** 10.1007/s00059-023-05210-5

**Published:** 2023-10-17

**Authors:** Anar Aghayev, Mattes Hinnerichs, Andreas Wienke, Hans-Jonas Meyer, Alexey Surov

**Affiliations:** 1 Department of Radiology and Nuclear Medicine, Otto von Guericke University, Magdeburg, Germany; 2 Institute of Medical Epidemiology, Biostatistics, and Informatics, Martin-Luther-University Halle-Wittenberg, Halle, Germany; 3 Department of Diagnostic and Interventional Radiology, University of Leipzig, Leipzig, Germany; 4 Ruhr-University-Bochum, Department of Radiology, Neuroradiology and Nuclear Medicine, Johannes Wesling University Hospital, Minden, Germany

**Keywords:** Epicardial adipose tissue, Computed tomography, Pulmonary embolism, Epikardiales Fettgewebe, Computertomographie, Lungenembolie

## Abstract

**Background:**

Epicardial adipose tissue (EAT) has been established as a quantitative imaging biomarker associated with disease severity in coronary heart disease. Our aim was to use this prognostic marker derived from computed tomography pulmonary angiography (CTPA) for the prediction of mortality and prognosis in patients with acute pulmonary embolism.

**Methods:**

The clinical database was retrospectively screened for patients with acute pulmonary embolism between 2015 and 2021. Overall, 513 patients (216 female, 42.1%) were included in the analysis. The study end-point was 30-day mortality. Epicardial adipose tissue was measured on the diagnostic CTPA in a semiquantitative manner. The volume and density of EAT were measured for every patient.

**Results:**

Overall, 60 patients (10.4%) died within the 30-day observation period. The mean EAT volume was 128.3 ± 65.0 cm^3^ in survivors and 154.6 ± 84.5 cm^3^ in nonsurvivors (*p* = 0.02). The density of EAT was −79.4 ± 8.3 HU in survivors and −76.0 ± 8.4 HU in nonsurvivors (*p* = 0.86), and EAT density was associated with 30-day mortality (odds ratio [OR] = 1.07; 95% confidence interval [CI]: 1.03; 1.1, *p* < 0.001) but did not remain statistically significant in multivariable analysis. No association was identified between EAT volume and 30-day mortality (OR = 1.0; 95% CI: 1.0; 1.0, *p* = 0.48).

**Conclusion:**

There might be an association between EAT density and mortality in patients with acute pulmonary embolism. Further studies are needed to elucidate the prognostic relevance of EAT parameters in patients with acute pulmonary embolism.

## Abstract (de)

**Hintergrund:**

Das epikardiale Fettgewebe ist mittlerweile bei koronarer Herzkrankheit als quantitativer bildgebender Biomarker als prognostischer Parameter etabliert. Ziel der vorliegenden Studie war es, diesen prognostischen Marker, der aus der Computertomographie-Pulmonalisangiographie (CPTA) abgeleitet wird, für die Vorhersage der Mortalität und Prognose bei Patienten mit akuter Lungenembolie zu untersuchen.

**Methoden:**

Retrospektiv wurde die klinische Datenbank im Hinblick auf Patienten mit akuter Lungenembolie zwischen den Jahren 2015 und 2021 analysiert. Es wurden 513 Patienten (216 Frauen, 42,1%) in die aktuelle Auswertung einbezogen. Studienendpunkt war die 30-Tage-Mortalität. Das epikardiale Fettgewebe wurde anhand der diagnostischen CTPA in semiquantitativer Weise gemessen. Dabei wurde das Volumen und die Dichte des epikardiale Fettgewebes für jeden Patienten ermittelt.

**Ergebnisse:**

Innerhalb der 30 Tage Beobachtungsdauer verstarben 60 Patienten (10,4%). Das mittlere Volumen des epikardialen Fettgewebes betrug 128,3 ± 65,0 cm^3^ bei den Überlebenden und 154,6 ± 84,5 cm^3^ bei den Nichtüberlebenden (*p* = 0,02). Bei den Überlebenden lag die Dichte des epikardialen Fettgewebes bei −79,4 ± 8,3 HU (Hounsfield-Einheiten) und den Nichtüberlebenden −76,0 ± 8,4 HU (*p* = 0,86). Die Dichte des epikardialen Fettgewebes stand mit der 30-Tage-Mortalität in Zusammenhang (Odds Ratio [OR] = 1,07; 95%-Konfidenzintervall [95%-KI]: 1,03; 1,1; *p* < 0,001), blieb aber in der multivariablen Analyse nicht statistisch signifikant. Zwischen dem Volumen des epikardialen Fettgewebes und der 30-Tage-Mortalität fand sich kein Zusammenhang (OR = 1,0; 95%-KI: 1,0; 1,0; *p* = 0,48).

**Schlussfolgerung:**

Möglicherweise besteht ein Zusammenhang zwischen der Dichte des epikardialen Fettgewebes und der Mortalität bei Patienten mit akuter Lungenembolie. Weitere Studien sind erforderlich, um die prognostische Bedeutung von Parametern des epikardialen Fettgewebes bei Patienten mit akuter Lungenembolie zu bestimmen.

Acute pulmonary embolism (PE) is a possible life-threatening cardiovascular disease with 30-day mortality rates ranging from 0.5% to over 20% depending on clinical symptoms at presentation [1, 2]. Yet, there are also low-risk clinical courses without severe complications and good clinical outcome [3]. An immediate risk stratification of patients with acute PE at the time of presentation is of great importance in order to characterize and identify patients at risk and to possibly escalate the treatment regimen [3].

Computed tomography pulmonary angiography (CTPA) is an established diagnostic modality in clinical routine [4–6]. It is considered the diagnostic gold standard for the diagnosis of PE, with a reported sensitivity and specificity up to 100% [4–6]. Most commonly, CTPA is performed directly at the time of the hospital admission to detect the PE [4]. Therefore, risk stratification based on CTPA could be very important [5]. There are already established imaging signs for the severe course of PE, which can be obtained via the CT images. Of these signs, the right-to-left ventricular diameter ratio has the strongest predictive value [5]. Another promising parameter is the contrast media reflux into the inferior vena cava [5, 7, 8].

Epicardial adipose tissue (EAT) is a type of visceral fat surrounding the myocardium and visceral layer of the pericardium. In certain conditions, EAT can secrete pro- and anti-inflammatory factors (e.g., adiponectin, interleukin [IL]-6, tumor necrosis factor (TNF)-α, and leptin) in the paracrine or endocrine pathways [9–11]. There is ample evidence that EAT is involved in the local regulation of myocardial and coronary function by modulating lipid metabolism and energy homeostasis. Clinically, the volume and thickness of EAT have been measured by cardiac magnetic resonance imaging (MRI), CT [9–11], and echocardiography [9–11]. As such, several studies have shown that enlarged EAT is associated with the occurrence and development of coronary artery disease [11]. The prognostic value of EAT was also evaluated in other diseases including Coronavirus disease 2019 [12]. However, it is unknown whether this parameter also holds prognostic information for patients with acute PE.

Therefore, the purpose of the present study was to investigate whether EAT is of prognostic relevance in patients with acute PE.

## Methods

### Patients

The present retrospective study was approved by the institutional review board of the University of Magdeburg (Nr. 145/21, Ethics Committee, Otto-von-Guericke University of Magdeburg, Magdeburg, Germany).

All patients with acute PE were retrospectively assessed within the time period 2015–2021. Inclusion criteria were:

- Sufficient CT images with clearly visible PE at the admission to hospital
- Available clinical data regarding clinical signs, serological parameters, and follow-up
- No thrombolysis before and/or during the CT acquisition

Exclusion criteria were:

- Severe image artifacts (i.e., due to implants or motion artifacts) as well as any form of treatment
- Missing clinical data/follow-up
- Thrombolysis before CT imaging
- Chronic PE

Overall, 513 patients (216 female, 42.1%) were included in the analysis. The mean age at the time of CT acquisition was 64.9 ± 15.6 years (median age: 66 years).

### Clinical parameters

The following clinical parameters were retrieved at the timepoint of hospital admission:

- Relevant clinical comorbidities (active malignant disease, surgery performed within the last 4 weeks, chronic lung disease, chronic heart failure)
- Blood pressure (mm Hg), heart rate (*n*/minute), need for intubation, need for vasopressor, need for intensive care admission
- The Simplified Pulmonary Embolism Severity Index (sPESI) score was calculated
- Mortality, assessed in number of days after diagnosis of PE

### Imaging technique

Computed tomography was performed at admission for every patient without any previous treatment. Diverse multislice CT scanners were used (Siemens Somatom Definition AS+, Siemens Healthcare, Erlangen, Germany, or Canon Aquilion Prime, Canon Medical Systems, Ottawara, Japan). In all cases, an intravenous administration of an iodinated contrast agent (60–150 mL Accupaque 300 mg/mL, GE Healthcare Buchler GmbH & Co. KG, Braunschweig, Germany; or Imeron 300, Bracco Imaging Deutschland GmbH, Konstanz, Germany) was given at a rate of 3.0–4.0 mL/s via a peripheral venous line. Automatic bolus tracking was performed in the pulmonary trunk with a trigger of 100 Hounsfield units (HU). Typical imaging parameters were 100–120 kVp, 25–200 mAs (tube current modulated 50–400 mA), slice thickness 1 mm, and a pitch factor of 1.4.

The right/left ventricular diameter was assessed for every patient in an axial slice.

### Epicardial adipose tissue

**Fig. 1:**
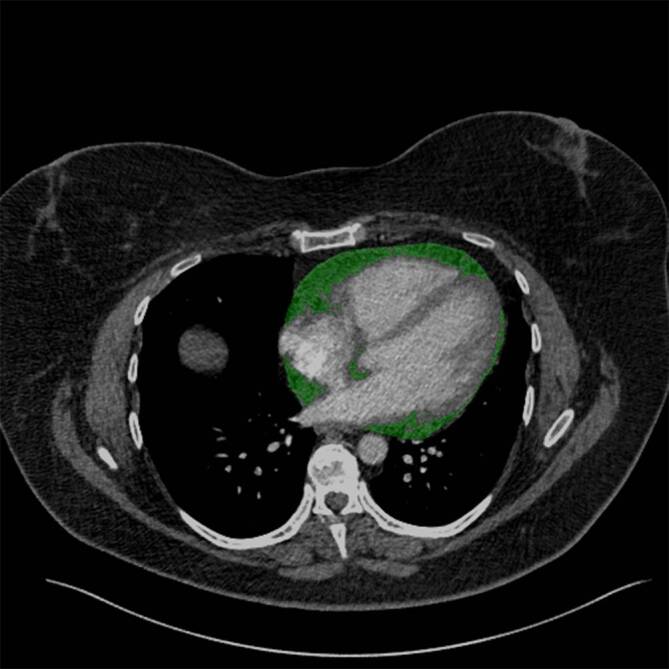
Representative case from the patient sample with segmental acute pulmonary embolism. The epicardial adipose tissue (EAT) segmentation is visualized with a *green overlay*. The EAT volume is 415.2 cm^2^ and the density is −82.7 HU

A trained radiologist (AA), blinded to patient outcomes, measured the EAT volume with a dedicated workstation using Intellispace Portal (Version 11; Philips, Amsterdam, The Netherlands). The EAT volume was calculated considering density values in the range between −30 and −190 HU for adipose tissue and respecting as anatomical limits the pulmonary artery bifurcation, the left atrium, and the aortic root as the upper limit and the diaphragm and the left ventricle apex as the lower limit; mean density in HU was also calculated. This was previously described in the literature [13]. The following parameters were calculated: EAT volume, density, and volume/body height. Figure 1 displays a representative case of our patient sample.

### Statistical analysis

The statistical analysis and graphics creation were performed using SPSS (IBM SPSS Statistics for Windows, version 225.0, IBM Corp., Armonk, NY, USA). Collected data were evaluated by means of descriptive statistics (absolute and relative frequencies). Group differences were calculated with the Mann–Whitney test and Fisher exact test, when suitable. Correlation analysis using Spearman’s test was carried out to elucidate associations between the parameters. Uni- and multivariable logistic regression analyses were employed to investigate the associations with 30-day mortality. In all instances, values of *p* < 0.05 were taken to indicate statistical significance.

## Results

Overall, 60 patients (11.7%) died within the 30-day observation period.

In survivors, the mean EAT volume was 128.3 ± 65.0 cm^3^ and in nonsurvivors it was 154.6 ± 84.5 cm^3^, *p* = 0.02. The density of EAT was −79.4 ± 8.3 HU in survivors and −76.0 ± 8.4 HU in nonsurvivors (*p* = 0.89; Table 1). Similar results were identified accordingly to hemodynamic stability of the patients (Table 2).

**Table 1:** Comparison between EAT parameters in survivors and nonsurvivors

Parameter	Survivors, M ± SD (*n* = 452)	Nonsurvivors, M ± SD (*n* = 60)	*p*
EAT volume (cm^3^)	128.3 ± 65.0	154.6 ± 84.5	0.02
EAT density (HU)	−79.4 ± 8.3	−76.0 ± 8.4	0.0048
sPESI Score	1.2 ± 1.0	1.7 ± 1.1	0.0001
Heart rate (1/min)	95.5 ± 23.3	104.8 ± 34.6	0.13
Systolic blood pressure (mmHg)	134.0 ± 27.3	129.0 ± 38.6	0.10
D‑dimer (mg/L)	5.2 ± 6.2	5.6 ± 5.7	0.81
Right/left ventricular diameter	1.1 ± 0.4	1.1 ± 0.5	0.48

*EAT* epicardial adipose tissue, *HU* Hounsfield unit, *M* mean, *SD* standard deviation, *sPESI* Simplified Pulmonary Embolism Severity Index

**Table 2:** Comparison between hemodynamic stable and instable

Parameter	Hemodynamic stable, M ± SD	Hemodynamic instable, M ± SD	*p*-value
EAT volume (cm^3^)	130.59 ± 66.31	143.16 ± 88.59	0.03
EAT density (HU)	−79.0 ± 8.4	−78.8 ± 8.8	0.89

*EAT* epicardial adipose tissue, *HU* Hounsfield unit, *M* mean, *SD* standard deviation

Results showed that EAT volume correlated with age (*r* = 0.17, *p* < 0.0001) and systolic blood pressure (*r* = 0.26, *p* < 0.0001). However, EAT volume did not correlate with troponin level (*r* = 0.07, *p* = 0.18), lactate level (*r* = −0.02, *p* = 0.73), and right ventricular diameter (*r* = 0.07, *p* = 0.08).

Furthermore, EAT density correlated with systolic blood pressure (*r* = −0.21, *p* < 0.0001), right ventricular diameter (*r* = 0.12, *p* = 0.0037), and troponin level (*r* = 0.17, *p* = 0.0021).

There were no correlations between right–left ventricular diameter and EAT volume (*r* = −0.06, *p* = 0.15) and EAT density (*r* = 0.04, *p* = 0.28).

Finally, EAT density was associated with 30-day mortality in univariable logistic regression analysis (OR = 1.06; 95% CI [1.01; 1.08], *p* < 0.001; Table 3). Furthermore, EAT volume did not influence 30-day mortality (OR = 1.0; 95% CI [1.0; 1.0], *p* = 0.48). After adjustment with the sPESI score, EAT density was not associated with 30-day mortality in multivariable analysis (OR = 1.0; 95% CI [1.0; 1.0], *p* = 0.38).

**Table 3:** Univariable regression analysis to predict 30-day mortality

	Univariable			Multivariable		
Parameter	OR	95% CI	*p*	OR	95% CI	*p*
EAT volume (cm^3^)	1.0	(0.89; 1.13)	0.87	–	–	–
EAT density (HU)	1.06	(1.01; 1.08)	< 0.001	1.0	(1.0; 1,0)	0.38
Gender	1.1	(0.70; 2.0)	0.50	–	–	–
Age	0.99	0.97; 1.009	0.34	–	–	–
Right/left ventricular diameter	1.00	1.0; 1.0	0.79	–	–	–
Heart rate	1.05	0.99; 1.01	0.34	–	–	–
Systolic blood pressure	0.99	0.98; 1.009	0.71	–	–	–
sPESI score	1.59	1.25; 2.05	0.001	1.59	1.25; 2.02	0.001

*EAT* epicardial adipose tissue, *HU* Hounsfield unit, *CI* confidence interval, *sPESI* Simplified Pulmonary Embolism Severity Index, *OR* Odds Ratio

## Discussion

The present study sought to establish the prognostic relevance of EAT quantified with density and volume in patients with acute PE.

Correct and rapid risk stratification can be crucial for patients with acute PE. According to clinical guidelines, an important factor for a massive or critical course is hypotension with a systolic blood pressure below 90 mm Hg [3, 14, 15]. However, the absence of hemodynamic instability does not exclude beginning with a possibly progressing right ventricular dysfunction [15]. A standardized anamnestic and clinical evaluation comprises the Geneva and Wells score as a first assessment [15]. Regarding laboratory biomarkers, elevated troponin concentrations are associated with a worse prognosis [15]. Elevated B‑type natriuretic peptide indicates right ventricular overload and is also associated with a worse prognosis in patients with acute PE [3, 15].

Echocardiography and CT can provide imaging information on right ventricular dysfunction but other reliable prognostic factors are still lacking to date [3, 15].

The prognostic and predictive implications of EAT have been extensively investigated in cardiovascular diseases, especially in coronary heart disease. The function of EAT in heart physiology includes its role in cardiac metabolism with mechanical protection of the coronary arteries, innervation, and potentially cryoprotection. However, recent evidence has revealed that EAT regulates multiple aspects of cardiac biology, myocardial redox state, and intracellular Ca^2+^ cycling [9–11]. It is noteworthy that electrophysiological and contractile properties of cardiomyocytes, and cardiac fibrosis, as well as atherogenesis are also regulated by EAT [9]. In a recent study of patients with diabetes, EAT volume was positively associated with age, BMI, pack-year history of smoking, and hypertriglyceridemia but negatively correlated with HDL cholesterol level [16].

Several studies elucidated a strong correlation between the severity of left ventricular diastolic dysfunction and the volume of EAT [17–19]. In acute PE, the prognostic relevance of EAT has not been systematically investigated until now.

The present analysis demonstrated some prognostic relevance of the density of EAT in patients with acute PE. The volume of EAT showed no association with 30-day mortality. Presumably, the inclusion of EAT into proposed risk scores such as the sPESI and the PEMS could increase the prognostic power of these scores [20].

The prognostic relevance of the density and not of the volume of EAT needs further consideration. The EAT volume was shown to be an important prognostic factor in patients with chronic coronary disease [9]. Yet, the quantification of the densities of adipose and muscle tissues is an emergent analysis, which might indicate earlier disease changes compared to the volume. For instance, there is recent evidence that muscle quality indicated by a decreased density of the muscle is an earlier finding of patients at risk compared to the muscle area [21–23]. Similar findings were reported for visceral adipose tissue in oncology patients [23]. Beyond that, in a recent study, the EAT density was also identified to be an important prognostic factor in patients with metabolic syndrome, showing better results when compared with EAT volume [24].

One important aspect of the present results is that EAT volume was statistically significantly different in the discrimination analysis, but did not remain significant in the logistic regression analysis. This could be interpreted as a possible signal that EAT volume could aid in the prediction of the prognosis but there might be a lack of statistical power in the present analysis.

It has to be acknowledged that manual EAT segmentation is a time-demanding procedure, which limits it translation into clinical routine. Yet, there are promising results that with the advent of artificial intelligence, new algorithms will be able to segment the EAT volume in a reliable manner [25]. This is a clear need for translation of EAT assessment of every patient with acute PET in clinical routine.

The present analysis is limited to a retrospective design with possible inherent bias. However, the EAT quantification was performed blinded to the clinical results in order to reduce possible bias. The present mortality rate of 10.4% is relatively high, which might be caused by selection bias. Moreover, it should be acknowledged that the present results might not be representative of patient samples with a lower case severity. Furthermore, the present study is based on a large cohort. To our best of knowledge, this is the first report on the associations between EAT and short-term mortality in acute PE.

## Conclusion

In conclusion, there might be an association between epicardial adipose tissue (EAT) density and mortality in patients with acute pulmonary embolism. Further studies are needed to elucidate the prognostic relevance of EAT parameters in patients with acute pulmonary embolism.

## Abbreviations

CT: Computed tomography
CTPA: Computed tomography pulmonary angiography
EAT: Epicardial adipose tissue
PE: Pulmonary embolism

## Declarations

All procedures performed in studies involving human participants or on human tissue were in accordance with the ethical standards of the institutional and/or national research committee and with the 1975 Helsinki declaration and its later amendments or comparable ethical standards. Informed consent was obtained from all individual participants included in the study. This research was supported by the German Federal Ministry of Education and Research (BMBF) as part of the University Medicine Network (Project RACOON, 01KX2021).
